# Toward Mitochondrial Targeting of Resistant Triple-Negative Breast Cancer Using Triphenylphosphonium-Conjugated Antimicrobial Peptides

**DOI:** 10.1021/acsptsci.5c00563

**Published:** 2025-10-07

**Authors:** Eda Kapan, Cemile Uslu, Haya Arab, Leen Ahmed, Rama Ali, Andrey G. Tereshchenkov, Natalia V. Sumbatyan, Alex Lyakhovich

**Affiliations:** † 52991 Sabanci University, Molecular Biology, Genetics and Bioengineering, Faculty of Engineering and Natural Sciences, Üniversite Caddesi No: 27, Orta Mahalle, Tuzla, İstanbul 34956, Turkey; ‡ Trinity College Dublin, The University of Dublin, College Green, Dublin 2, Dublin D02 PN40, Ireland; § Lomonosov Moscow State University, A.N. Belozersky Institute of Physico-Chemical Biology, Moscow 119234, Russia; ⊥ Lomonosov Moscow State University, Department of Chemistry, Moscow 119991, Russia

**Keywords:** antimicrobial peptides, cancer resistance, cancer stem cells, mitochondria, oxidative phosphorylation, mitophagy

## Abstract

Metastatic evolution of malignant tumors following standard anticancer therapies and the emergence of resistant cancer cell populations remain major challenges in oncology. One promising strategy is to develop compounds that selectively target mechanisms of therapeutic resistance. Unlike therapy-sensitive malignant cells, which rely primarily on glycolysis for energy, many chemoresistant cells and cancer stem cells (CSCs) preferentially utilize mitochondrial oxidative phosphorylation (OXPHOS). In this study, we employed a triple-negative breast cancer model to demonstrate that short antimicrobial peptides can significantly suppress the metastatic potential of resistant cancer cells and reduce the formation of CSC-like mammospheres by disrupting mitochondrial respiration. This effect was further enhanced by conjugating the peptides to the mitochondrial-targeting cation triphenylphosphonium (TPP). Mechanistic studies revealed that these compounds induce oxidative stress and mitophagy and suppress mitochondrial translation. Collectively, these findings suggest that TPP-conjugated peptides represent a promising therapeutic strategy for targeting OXPHOS-dependent resistance in aggressive solid tumors.

## Graphical abstract

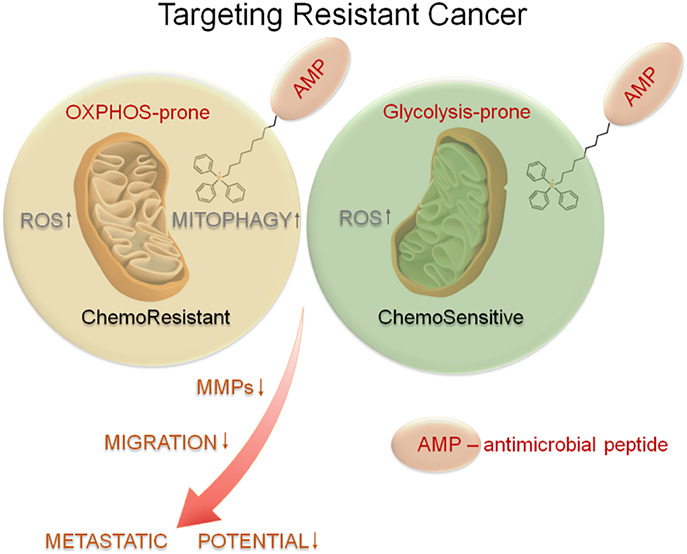

Contrary to the stereotype, cancer patients do not die from cancer as such, but from metastases—the spread of malignant cells from the primary tumor to other parts of the body.^1^ Most often, such processes are a consequence of tumor evolution and take quite a long time. Attempts to stop metastasis after the surgical stage include traditional treatments—chemo-, radio-, or immunotherapy, which very often lead to tumor resistance.^2^ Essentially, rapidly dividing cells respond to treatment, while slowly dividing cells mutate and, in accordance with Darwinian evolution, give rise to more aggressive clones from which metastasis occurs.^3^ The mechanisms of such cancer resistance are diverse, ranging from activation of specialized efflux pumps that remove drugs from the cell to enhancement of the DNA repair response.^4–6^ Moreover, resistant tumors may include a subpopulation of cancer stem cells (CSCs) that possess stemness quality and are thought to be responsible for metastatic disease.^7^ Therefore, exploring new approaches and finding new compounds to specifically target a population of resistant cancer cells or CSCs are of undoubted interest for anticancer therapy.

Recently, antimicrobial peptides (AMPs), discovered first in soil bacilli^8^ and then in mammals,^9–11^ have been used not only as microbicidal or cytolytic compounds,^12^ but also as potential anticancer agents due to their ability to induce malignant cell death.^13–17^ AMPs are small bioactive proteins that are components of the body’s innate immune system.^9^ The mode of action of AMPs is not yet fully elucidated, but it is known that peptides such as latampulin, allicin, and dermaseptin-PS1 can alter cell membrane permeability, leading to cellular stress and apoptosis.^18^ Studies also show that AMPs such as LL-37 and saraflagin can successfully target resistant cancer cells, including melanoma^19^ and colorectal carcinomas. Lactoferricin B is cytotoxic to neuroblastoma cells.^20^ Other specific AMPs are able to activate the immune response and attack tumor cells.^21^ Interestingly, in cancer cells AMPs can affect mitochondria, disrupting their normal function, which can lead to cell death.^22,23^

In our recent studies, we have shown that many types of malignant tumors have OXPHOS-dependent chemoresistant cells and therapy-sensitive cancer cells that prefer glycolysis (Warburg effect).^24,25^ This has led us to propose the discrimination of resistant cells by inducing mitochondrial dysfunction.^26–29^ Since mitochondria have the property of alphaproteobacteria, it has been suggested to induce mitochondrial dysfunction using antimicrobials active against Gram-negative bacteria, such as AMP bactenecin 7 (Bac7) or its derivatives, synthesized by ribosomes in granulocytes and possessing a nonlytic mechanism of action on bacteria associated with translation inhibition.^30–33^ However, the wider use of AMPs as anticancer drugs has its limitations due to the short lifespan of such peptides in the cell.^34,35^ In this regard, a number of conjugates, in particular triphenylphosphonium (TPP), have been proposed to stabilize short peptides.^36,37^ In addition, TPP is often used to deliver probes to organelle membranes.^38^ To enhance drug delivery to mitochondria and reduce the overall cytotoxicity of antimicrobials to healthy cells, we have recently utilized conjugates of TPP with chloramphenicol as a possible anticancer compound.^39^

In the present work, we applied newly synthesized TPP-AMP conjugates^40^ to target resistant cancer cells of triple-negative breast cancer (TNBC) as the most difficult-to-treat form of breast cancer. We were able to show that some TPP-AMPs specifically inhibit the growth of chemoresistant TNBC cells as well as the formation of mammosphere—breast CSC analogs. This is accompanied by increased oxidative stress *in vitro* and *in vivo*, induction of mitochondrial dysfunction, triggering of selective autophagy (mitophagy), and partial inhibition of mitochondrial protein synthesis. Among other findings, we recognized decreased activity of metalloproteinases, specifically in chemoresistant TNBC cells. Overall, these conjugated peptides appear to be a promising direction in oncology when used to treat resistant solid tumors.

## Materials and Methods

### Cell Lines and Treatment

MDA-MB-468 and MDA-MB-231 commercial cell lines were purchased from ATCC and authenticated in Ana Janec’s lab (UPF, Barcelona). Human dermal fibroblasts were gifted by Nur Mustafaoglu’s lab. Cells were cultured in Dulbecco’s Modified Eagle’s Medium supplemented with 10% FBS, 1% Sodium Pyruvate and 1% l-glutamine. Chemoresistant cell lines were established with continuous treatment for 6 months with escalating doses of anticancer therapeutic agents, such as cyclophosphamide and cisplatin. To maintain resistance, the relevant cells were always cultured in the presence of the relevant drugs at IC5 doses. Experiments related to the measurement of respiration, ATP levels, and activities of mitochondrial complexes were performed in the media containing no antibiotics.

### Chemicals and Synthesis of AMP and Conjugates

All reagents used were of analytical grade or the highest grade available. AMP synthesis and TPP conjugation was performed essentially as described earlier.^40^ Briefly, peptides were synthesized according to the standard Fmoc/Pbf­(tBu) solid-phase peptide synthesis protocol using 2-chlorotrityl chloride resin and HBTU/DIPEA activation. The Fmoc protective group was removed with a piperidine solution. TFA along with scavengers (reagent K) was used for the cleavage of peptides from the resin and the removal of side-chain protective groups. TPP-C_10_-AMP was synthesized directly on the resin by the condensation of the (10-carboxydecyl)­triphenylphosphonium bromide (TPP-C_10_-COOH) with a peptidyl polymer. TPP-C_10_-COOH was synthesized from TPP and 11-bromoundecanoic acid as described in ref. 41. AMP-C_10_-TPP was obtained via the conjugation of the protected peptide with (10-aminodecyl)­(triphenyl)­phosphonium bromide (NH_2_–C_10_-TPP) using HBTU as an activating agent. Fmoc/Pbf/tBu-protected peptide was cleaved from the resin using HFIP. NH_2_–C_10_-TPP was obtained in two stages via the conjugation of TPP with dibromodecane at 85 °C for 72 h, according to ref. 42, and the amination of the resulting product with 7 M ammonia in methanol at 85 °C for 4 h.^43^ The compounds were purified on silica gel or via HPLC and analyzed using the LC-MS, NMR, and HR-MS techniques.^40^

### Cell Viability Assays

Stock solutions of the test compounds (50 mM) were prepared in DMSO, and each compound solution was diluted in the cell medium to reach the desired well concentration. The concentration of DMSO per well was always lower than 0.1%. The cells were exposed to the test compounds at three increased concentrations for 24 h, and cellular viability was evaluated by the MTT color change compared to control untreated cells (% of control, *n* ≥ 4). For the Trypan Blue (TB) exclusion test cells were trypsinized after treatment with the drugs, and aliquots of 2 × 10 μL cells in PBS were mixed with 20 μL of 0.4% TB solution diluted in PBS. Cells were immediately counted under the microscope using a hemocytometer, and the number of TB stained (dead cells) and the total number of cells were used to calculate cell viability: % cell viability = (total number of cells – number of dead cells)/total number of cells × 100.

### Cell Adhesion Assay

Cell adhesion assay was performed as described previously.^39^ Shortly, cells were treated with the corresponding compounds for 24 h, washed in PBS, and counted, and an equal number of cells were plated in 24-well plates allowing them to attach to the surface. After 30 min of agitation on a shaker, each plate was washed with PBS until no floating cells remained and then cross-linked with 4% paraformaldehyde for 10 min, replaced with the fresh PBS, stained with crystal violet for 5 min, washed 3 times with PBS, and dried. Stained cells were dissolved in 1% SDS and DMSO/ethanol mixture (50/50 v/v), and absorbance was measured at 570 nm with an ELISA plate reader. The experiment was repeated three times, and for each dish, four wells were scored.

### Migration (Wound Healing) Assay

The assay was performed essentially as described earlier.^44^ Briefly, cells were grown to 80–85% confluence on a u-slide (ibidi GmbH, Germany) in the presence or absence of AMPs, and a “wounding” line appeared after plastic inserts were removed from the cell monolayer. The width of the wound was measured under a microscope after 1, 8, 16, and 36 h to assess the migration ability of the cells. In parallel, the antiproliferative reagent mitomycin C (MMC, 0.0002 mg/mL) was added to discriminate effects on proliferation and migration. Results were analyzed with the Student’s *t* test.

### Colony Formation Assay

The assay was performed essentially as described previously.^6^ Shortly, cells were suspended in colorless DMEM media containing 0.2% agarose in the presence or absence of AMPs, and layered in triplicates or quadruplicates over a solid base of 0.6% agarose in 6-well plates. Cells were incubated at 37 °C for 2 weeks and the average number of colonies (<30 cells each) per well was counted.

### Tumorsphere Formation

To obtain cancer stem-like cells (mammospheres), we followed a previously published protocol.^39^ Shortly, a single suspension of corresponding TNBC cells was prepared using enzymatic disaggregation (1× Trypsin-EDTA, Gibco, 25300062), and the cells were plated at a density of 10,000–12,000 cells/mL in a Cancer Stem Cell medium (C-28070, PromoCell, Heidelberg, Germany) containing 10 nM FGF and 10 nM EGF (all from Sigma) in 3-times poly-2-hydroxyethyl methacrylate (Poly-HEMA, Santa Cruz Biotechnology, Dallas, TX, USA, sc-253284)-coated plates. Cells of the first generation (G1) were collected 6 days after seeding. For experiments, the second generation of tumorspheres was treated with AMPs or DMSO (control), followed by the above-mentioned procedure of tumorsphere formation. The relative numbers of 3d-generation (3G) tumorspheres per 4 squares were counted manually. The experiments were performed independently at least 2 times, with several replicates.

### Gelatin and *In Situ* Zymography

Gelatinase activity was evaluated by zymography as described before.^44^ Aliquots of media were resolved on a 10% SDS-polyacrylamide gel containing 0.1% gelatin under nonreducing conditions. Following electrophoresis, the gels were washed twice with 2.5% Triton X-100 for 30 min to remove SDS and renature the MMP species in the gels. The gels were then incubated in developing buffer (10 mM Tris–HCl pH 7.5, 0.1% Triton X-100, 1 mM CaCl_2_) overnight to induce gelatin lysis by renatured MMPs. The active MMPs were detected as clear bands. Equal loading was controlled by staining a regular polyacrylamide gel with corresponding probes.

### Plasmid Transfection and Detection of LC3 Foci for Mitophagy

The plasmid pRFP-LC3 was purchased from AddGene and transfection was performed as in early studies^45^ using Lipofectamine 2000 (Thermo Fisher) according to the manufacturer’s instructions. The same day of transfection, AMPs were added to the cell media, and 2 days after, the RFP-LC3 puncta staining was determined by fluorescence microscopy (Nikon 1 AR inverted Microscope, × 40 objective, 561 nm excitation length). Twenty-five cells were analyzed, and the average number of RFP-LC3 puncta per cell was counted. To detect mitophagy events, cells were costained with 1 μM MitoTracker Green reagent, and the average number of colocalized green mitochondrial and LC3-red foci was calculated and plotted as a % on bar diagram.

### Mitochondrial Membrane Potential (Δψm)

JC-1 staining was performed according to the manufacturer’s protocol (Thermo Fisher, #T3168). Cells (1 × 10^6^) cultured for 24 h in 6-well plates and treated with ATP-conjugated compounds for 3 days were harvested, washed with PBS, and resuspended in 1 mL PBS. JC-1 stock solution was prepared in DMSO at a concentration of 5 mg/mL. JC-1 dye was then added to the resuspended cells at a final concentration of 2 μg/mL followed by incubation at 37 °C, 5% CO_2_ for 30 min. After incubation, the cells were washed with PBS, plated into 96-well plates (1 × 10^4^ per well) in 100 μL of transparent media, and analyzed spectrofluorimetrically. The average red and green intensity values in each biological replica were determined and the red and green intensity ratio for each was calculated followed by a Student’s *t* test (*n* = 4). As a control, carbonyl cyanide 3-chlorophenylhydrazone (CCCP) was added at a concentration of 0.2 μM 6 h prior to JC-1 staining.

### Analysis of Mitochondrial Mass Content

Mitochondrial mass content was determined as previously described.^45^ Briefly, 1 × 10^6^ cells were seeded and incubated at 37 °C and 5% CO_2_ for approximately 24 h, after which the cells were stained with MitoGreen (1 μM final), washed with PBS, resuspended in 100 μL PBS, and analyzed spectrofluorimetrically by determining the mean green intensity value in each well relative to the unstained control. The ratio of signal intensities for each sample was then calculated and the mean value for all 3 biological replicates was plotted.

### Intracellular and Mitochondrial ROS

Both intracellular and mitochondrial ROS measurements were performed based on our early established procedures.^26^ Measurements of intracellular ROS were based on the ability of cells to oxidize the fluorogenic dye 2,7-dichlorofluorescein (H2DCF-DA) to their corresponding fluorescent analogues that allowed ROS determination in living cells. Mitochondrial ROS was detected by measuring mitochondrial superoxide anion with MitoSOX red reagent according to the manufacturer’s protocol (Invitrogen, Thermo Scientific, M36008) determining the mean red intensity value in each well relative to the unstained control. The signal intensities for each sample were calculated, subtracted from the control of autofluorescence samples, and the mean value for 3–5 biological replicates was plotted on the graph.

### *Artemia salina* Maintenance for *In Vivo* Drug Testing

For the toxicity and mtROS assay, the maintenance and hatching of *A. salina* was designed as recently described.^46^ Treatment with AMP conjugates was done on day 3 after cyst hatching. Shortly, the dry cysts (1 g of Ocean Nutrition, batch number: 0N13280) were incubated in a well-aerated (air pump) 1.5 L aquarium with a thermostat submerged into artificial salty water (17 g/L Tropic Marin Pro Reef Sea Salt). The aquarium was placed on a magnetic stirrer with 300 rpm, and the thermostat was set at 28 °C for a 14 h light/10 h dark photoperiod. On the third day, the stirrer was stopped in order for the eggs to settle down for 15 min and fresh nauplii were collected by positive phototaxis using light to direct them to a specific region followed by distribution across 6 × 50 mL burets covered with aluminum foil leaving the bottom exposed to light and left for 2 h in the presence or absence of AMPs. Approximately 1 mL from every buret was poured into a single 50 mL Falcon tube to allow for the manual counting of nauplii under a light microscope (average range between 800 and 1000 species). For mitochondrial ROS levels, the same procedure was performed in Vi-Cell viability analyzer after staining with mitochondrial superoxide indicator MitoSOX Red followed by fluorescence measurement on a fluorescence plate reader at the excitation/emission wavelengths of 510/580 nm. Viability was determined manually.

### Measurement of ATP Level

ATP level was measured using the ATPlite kit as described in the manufacturer’s manual (PerkinElmer, Spain, 6016943). A solution of 10,000 cells in 100 mL of media/well was plated in triplicates in a black 96-well plate with a clear bottom. 50 μL of reagent was added to each well, and the plate was mixed for 5 min on an orbital shaker to induce cell lysis followed by incubation in the dark for 10 min to stabilize luminescence. The ATP content was then measured with Biotek’s Synergy Mx luminometer.

### Oxygen Consumption Rate (OCR) Measurement and Mitochondrial Profiling

To measure the OCR, the Seahorse XFe-24 analyzer (Agilent Technologies Spain, S.L.) was used. In short, 50,000 cells per well were seeded in triplicate or quadruplicate into XFe 24-well plates and treated with AMPs at IC20 concentrations. After 72 h of treatment, cells were washed with PBS and prewarmed XF assay media (Agilent, 102353-100), supplemented with 5.5 mM glucose, 2 mM pyruvate, and 2 mM l-glutamine was added to each well. Cells were then maintained at 37 °C in a non-CO_2_ incubator for 1 h. The Cell Mito Stress Test kit was used to measure mitochondrial parameters by an XF24 Analyzer. Measurements were normalized with a posterior BCA assay for total protein concentrations.

### Western Blotting and Sample Preparation

Analyses were performed essentially as described in Kumari et al.^47^ Samples were equilibrated for protein using a BCA assay, and lysates were separated on 7.5% or 4–20% acrylamide gels, blotted on nitrocellulose membranes, and incubated overnight with the appropriate primary antibodies: β-actin, p62 (no. 8025), LC3A/B (no. 4108), PINK1 (D8G3, no. 6946), Parkin (no. 2132), and VDAC (no. 4866) (all from Cell Signaling, Spain) followed by detection with corresponding HRP-conjugated secondary antibodies (Sigma).

### Statistical Analysis

All experiments were performed independently at least three times with 2 or 3 replicas. Unless otherwise stated, 2-way ANOVA (multiple comparisons) was applied utilizing the GraphPad Prism software version 8.0.1. *p* < 0.05 was considered as significant.

## Results

### AMPs Specifically Inhibit Proliferation of Resistant TNBC Cells

To better explore the mechanisms linking chemoresistance and OXPHOS, we generated models of MDA-MB-231 cells (invasive, mesenchymal phenotype) resistant to cisplatin and MDA-MB-468 cells (a more epithelial-like phenotype known to respond differently to alkylating agents) resistant to cyclophosphamide, the two commonly used anticancer drugs in clinical practice.^29^ By using different chemotherapeutics for each TNBC subtype, we aimed to model diverse resistance mechanisms that better reflect the heterogeneity of TNBC in clinical settings.

Our recently published and unpublished (Figure S1) data indicate increased oxygen uptake, ATP synthesis, mitochondrial mass, and expression of OXPHOS proteins in chemoresistant cells relative to their chemosensitive counterparts.^28^ Since the MTT assay depends on mitochondrial activity and this could affect the accuracy of the experiments, we measured cell proliferation in parallel by directly counting of unstained (live) and Trypan Blue-stained (dead) cells and showed that both methods give similar results (Figure S1C,D). In addition, TNBC patients show a poor clinical prognosis when the levels of the OXPHOS genes are overexpressed (Figure S1F). All this suggests that chemoresistant OXPHOS-predisposed TNBC cells exhibiting enhanced mitochondrial functions can be a legitimate target for anticancer treatment. Since Gram-negative bacteria resemble eukaryotic mitochondria, we and others earlier proposed using specific antimicrobials to target mitochondrial OXPHOS.^28,48–50^ Here we assayed TNBC cells over AMPs previously shown activity against Gram-negative bacteria^40^ and representing short peptides with/without a TPP moiety conjugated via an alkyl linker either with C- or N-terminus (Figure 1A). Our results demonstrate that TPP-conjugated AMPs preferentially inhibit chemoresistant TNBC cells and much less corresponding sensitive cancer cells (Figures 1 and S2).

**Figure 1:**
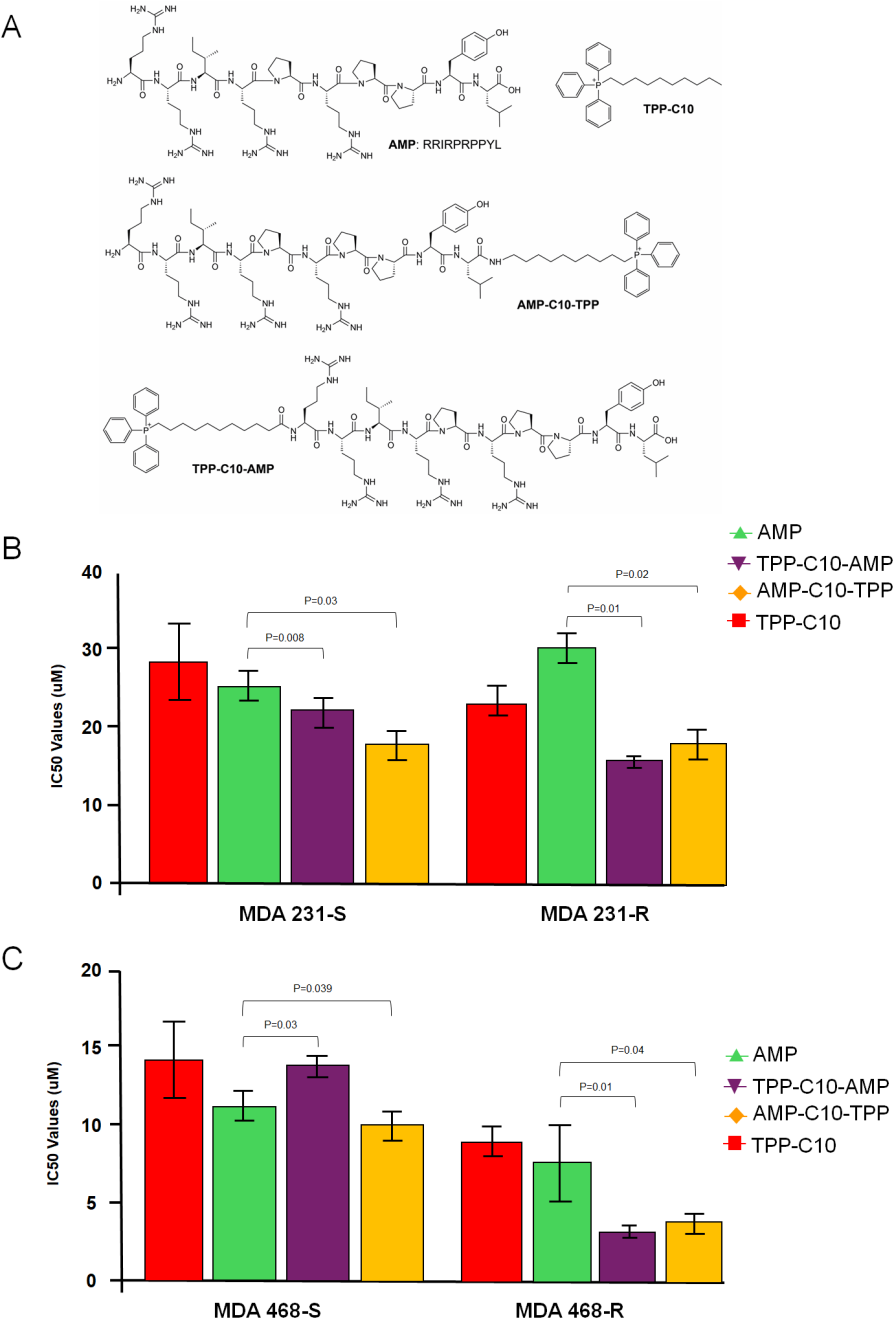
AMP conjugates preferentially inhibit chemoresistant TNBC cells. (A) Shown are the formulas of AMPs and corresponding conjugates used in this study. (B) MDA-MB-231 and (C) MDA-MB-468 TNBC cells, sensitive (S) or chemoresistant (R) to cisplatin or cyclophosphamide, were assayed over an increased concentration of TPP-C10-AMP (full data are shown in Figure S2) and IC50 values were plotted on the graph. All results are representative of at least three independent experiments with at least three replicas per treatment point. The exact IC50 values are provided for each graph. Data indicate the mean ± SEM, *n* = 4.

### AMP-TPPs Induce Preferential Mitochondrial Dysfunction of Chemoresistant TNBC Cells

Since OXPHOS is important not only for targeting resistant cancer cells but also affects the bioenergetics of healthy cells, we first showed that the selected AMP conjugates have virtually no effect on the proliferation of noncancerous cells (fibroblasts) compared to cancer cells up to IC50 concentrations (Figure S3). For the remaining experiments, we used IC20 concentrations. We tested the ability of AMP conjugates to induce mitochondrial dysfunction in our TNBC models. To this end, we first measured key mitochondrial parameters by performing the Seahorse experiment (Figure 2A–D).

**Figure 2:**
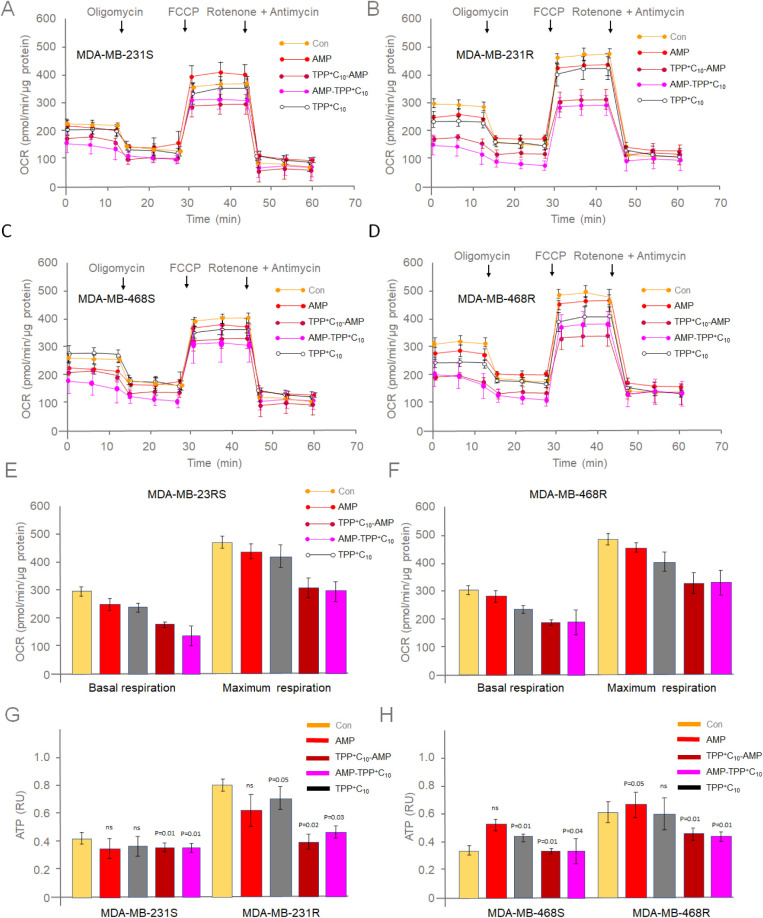
AMP-TPPs cause mitochondrial dysfunction of chemoresistant TNBC cells. Mitochondrial profiling was performed in both TNBC-sensitive (A,C) and resistant (B,D) cells following treatment with corresponding AMPs or controls. Cellular oxygen uptake followed by the addition of oligomycin (inhibits ATP synthase) was performed to study basal respiration, while the electron uncoupler carbonyl cyanide-4-(trifluoromethoxy) phenylhydrazone (FCCP) was added to measure maximal respiration (E,F). ATP levels were measured using the ATPlite kit (G,H). Experiments were performed independently over three times. Values are reported as means ± SD (*n* ≥ 3). *p < 0.05 compared with the control group.

Our results indicate that both basal and maximal respiration were presumably decreased in chemoresistant TNBC cell lines after AMP-TPP treatment (Figure 2E,F). This occurred in parallel with a statistically significant decrease in ATP levels (Figure 2G,H) and did not affect the mitochondrial content (Figure S4A,B). Unconjugated AMP did not significantly alter mitochondrial function, but the TPP ligand itself was able to depolarize the mitochondrial membrane, as revealed by measuring the mitochondrial membrane potential (ΔΨm) before and after treatment (Figure S4C,D). Taken together, these results suggest that AMP-TPP specifically induces mitochondrial dysfunction in chemoresistant TNBC cells by suppressing OXPHOS.

### AMP-TPP Conjugates Preferentially Reduce the Metastatic Potential of Chemoresistant TNBC Cell Lines

Cancer cells with high metastatic potential possess several distinct characteristics that allow them to enhance motility, survive in anchorage-independent conditions, and colonize distant organs. We were unable to see statistically significant changes in cell adhesion upon exposure of cells with moderate (IC20) concentration of AMP conjugates (Figure S5). However, the results showed a decrease in migration of resistant compared to sensitive TNBC cells after treatment with AMP-TPP conjugates but not with AMP or TPP (Figure 3A–D). In order to distinguish the effects of TPP conjugates on cell migration from cell proliferation, we examined wound closure in the presence of the antiproliferative compound MMC, which stops DNA replication, ensuring that the observed gap closure reflects true migration. In the presence of MMC, resistant cells demonstrated a more pronounced suppression of migration when treated with TPP conjugates (Figures 3E,F and S6). This sensitivity to migration inhibition may indicate suppression of the metastatic potential of resistant cells, although it does not account for a possible increase in DNA repair in resistant cancer cells relative to sensitive ones.

**Figure 3:**
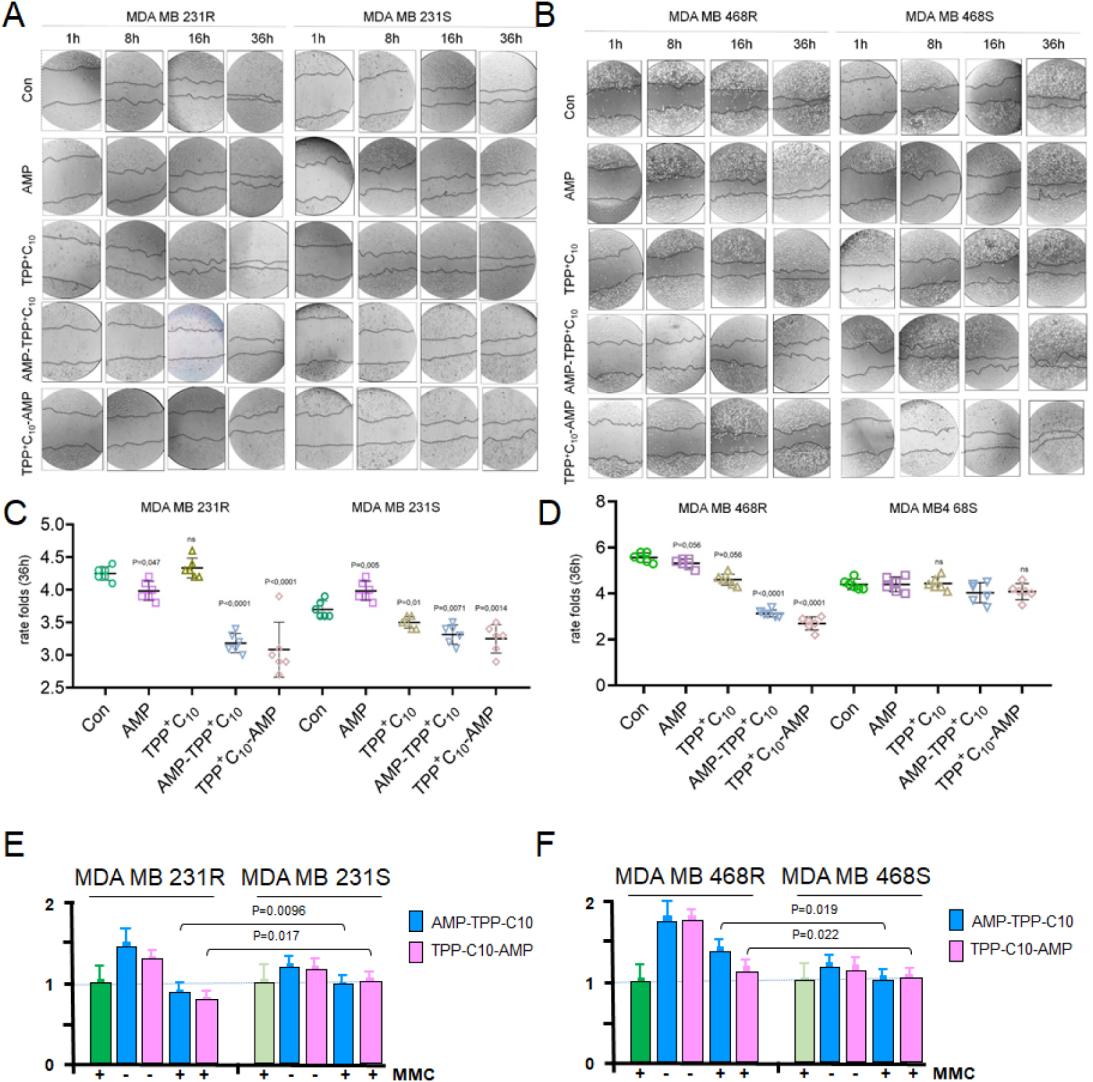
AMP-TPPs reduce migration of chemoresistant TNBC cells independently of cell proliferation. MDA-MB-231 (A,C) and MDA-MB-468 (B,D) TNBC cells in 6-well plates at a cell density of 80% were treated with IC20 of the respective compounds for 1 day and migration was assessed. Images were taken 1, 8, 16, and 36 h after scratch application (top), and the gap-filling rate was calculated and plotted on the graph diagrams (bottom). For each time point, at least 3 replicates were counted, each with 3 distance measurements. (E,F) Same as above assay was performed in the presence of the antiproliferative compound mitomycin C (0.0002 mg/mL, MMC), which blocks DNA replication. Migration was assessed after 36 h after scratch application and the gap-filling rate was calculated and plotted on the graph. Untreated control + MMC is used as the base reference point (normalization = 1.0) for all other conditions (Figure S6).

Next, the colony-forming ability was performed (Figure 4A–D). The results showed a decrease in colony formation of chemoresistant compared to sensitive TNBC cells after treatment with AMP-TPP conjugates, but not with AMP or TPP. Another feature of increased metastatic capacity is the enhancement of epithelium to mesenchyme transition (EMT), which is often accompanied by increased activity of matrix metalloproteases (MMPs). To test whether the selected AMP-TPPs could affect MMPs, we performed zymogram analysis and not only demonstrated that chemoresistant TNBC cells secrete more active MMPs but also showed that AMP-TPPs were able to suppress this activity (Figure 4E,F). Altogether, these results show that AMP-TPPs can specifically reduce the tumorigenic capacity of chemoresistant TNBC cells.

**Figure 4:**
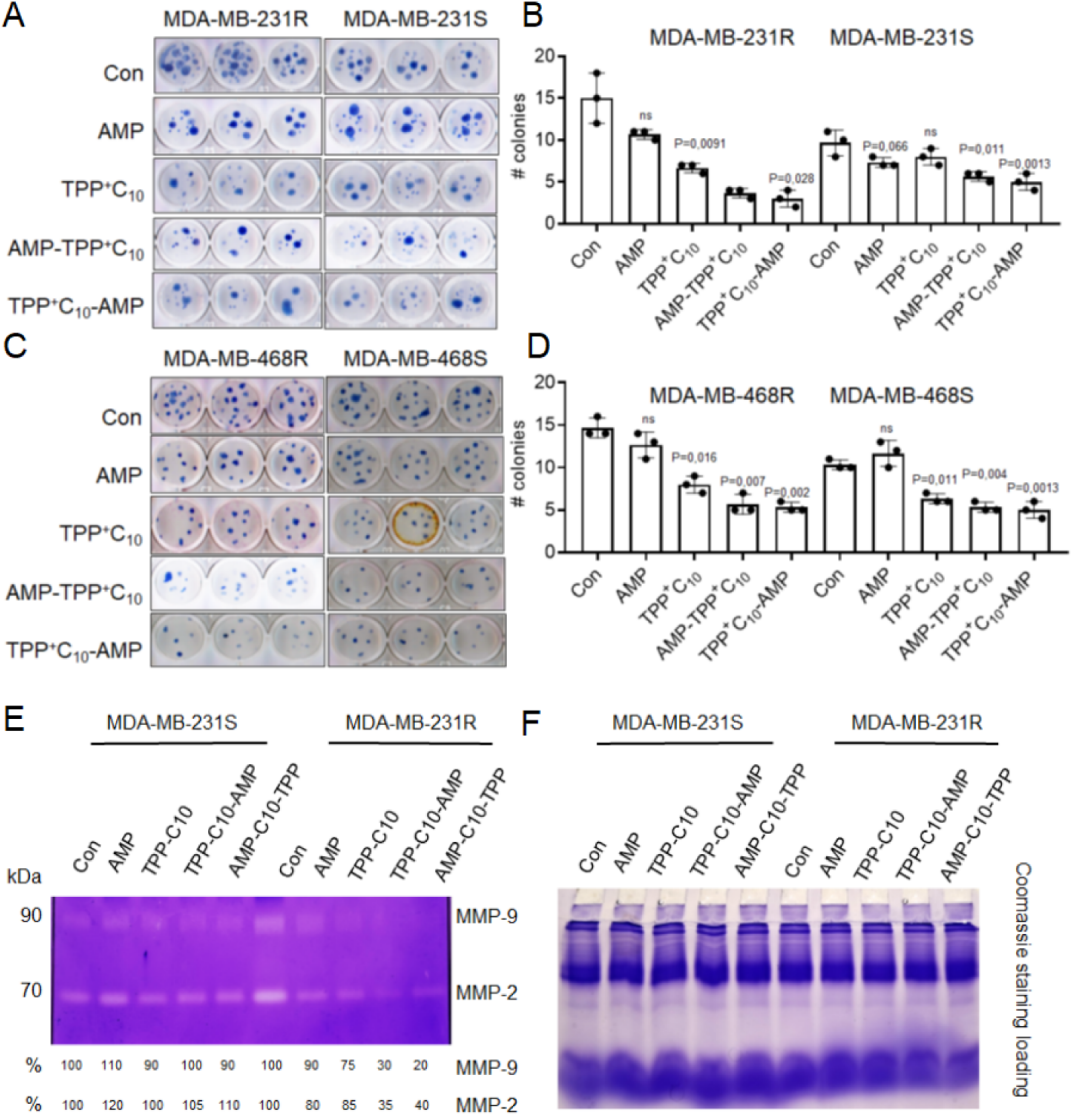
AMP-TPPs reduce the metastatic properties of TNBC cells. The effect of AMP conjugates (all at IC20) on colony growth of MDA-MB-231 (A,B) and MDA-MB-468 (C,D) TNBC cells over 3 weeks (*n* = 4). The top row shows examples of stained colonies. Those with more than 30 cells were counted. All values are presented as mean ± SD (*n* ≥ 3) compared to the respective control groups. (E,F) *In situ* zymography for chemosensitive (S) and chemoresistant (R) MDA-MB-231 cell media exposed to corresponding AMPs for 2 days (all IC20). Representative images show MMP gelatinase activity of corresponding samples. Data are representative of more than two independent experiments and the average values are shown on the bottom.

### AMP-TPP Conjugates Disrupt the Formation of CSC-like 3D Mammospheres in Chemoresistant TNBC Cell Lines

Often, the tumorigenic ability of cancer cells can be determined by the formation of 3D mammospheres emulating the CSCs, which are associated with cancer resistance and the ability of primary tumors to metastasize. To this end, we established multigeneration models of CSC-like mammospheres (Figure 5A) and examined the effect of AMP compounds by counting the average number of 3D generation mammospheres (Figure 5B). First, we showed that resistant TNBC cells produced a higher number of mammospheres on average, indicating their pronounced metastatic potential. Exposure of the conjugates to the cellular environment showed that AMP-TPP reduced the number of mammospheres produced from chemoresistant cells but less from sensitive TNBC cells (Figure 5C,D). Overall, these results suggest that AMP-TPP conjugates can specifically reduce the tumorigenic potential of chemoresistant cancer cells.

**Figure 5:**
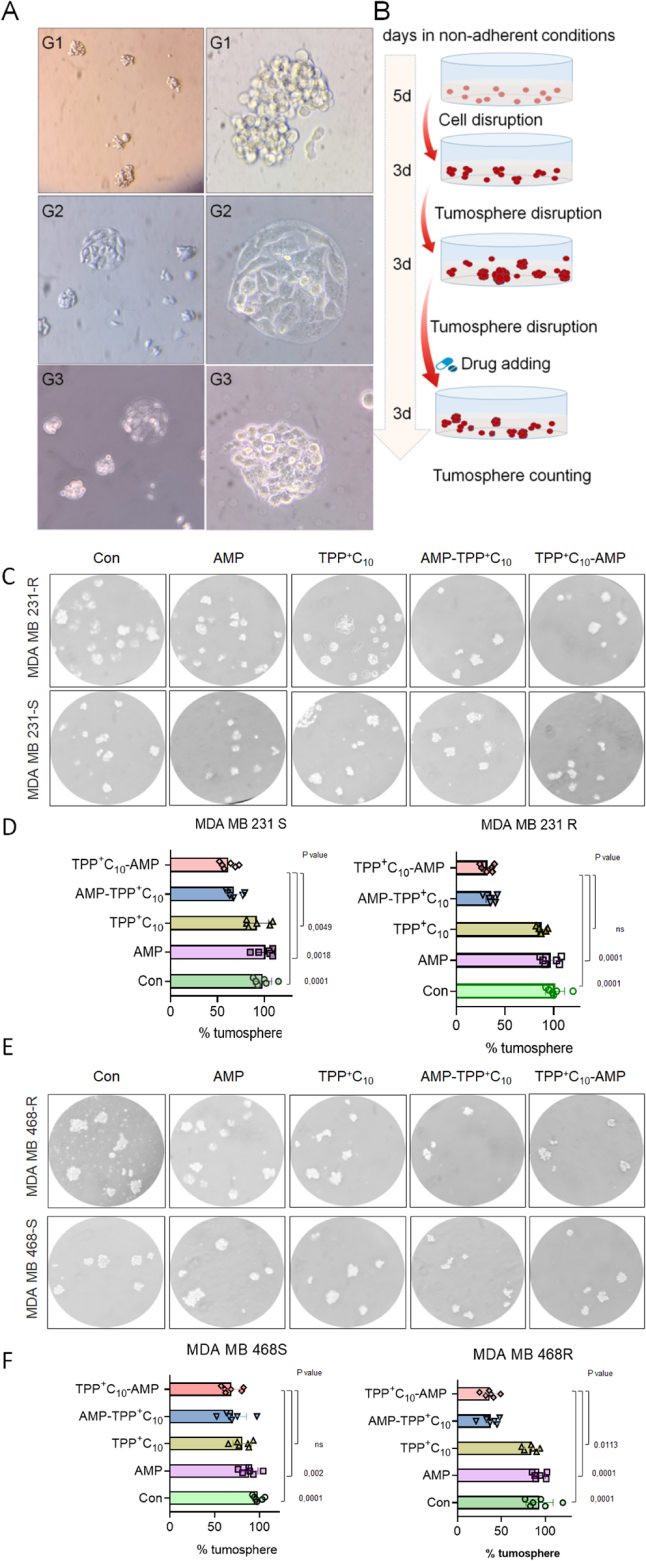
AMP-TPPs inhibit the formation of CSC-like cells. (A) Representative images of 3D mammospheres up to 3 generations and (B) the corresponding experimental design. Formation of third-generation spheroids from sensitive and chemoresistant TNBC cells was performed under nonadhesive conditions for 3 days in the absence or presence of the respective conjugates (IC20). (C,E) Representative images of treated cells show a reduction in the number of spheroids. (D,F) Results represent the mean of 5 independent experiments. Data are reported as mean ± SEM. The *p*-values, all relative to the control, were statistically significant.

### AMP-TPP Conjugates Induce Oxidative Stress, Mitophagy, and Inhibit Mitochondrial Protein Synthesis

Our previous data on antibactericidals demonstrated the enhancement of oxidative stress as one of the mechanisms of their killing effects.^29^ Here, we verified that the selected peptide conjugates increased mitochondrial ROS production, especially in chemoresistant TNBC cells (Figure 6A,B). To test whether these compounds can induce a similar increase in ROS in noncancer models, we performed *in vivo* experiments on our previously used *A. salina* model^46^ and showed a corresponding increase in mtROS, with no change in organism viability (Figure 6C,D). This may indicate that AMPs can be used against tumorigenic cells without affecting noncancerous cells. Since ROS accumulation can trigger selective autophagy to eliminate mitochondria with mitochondrial dysfunction,^51,52^ we tested the role of AMP conjugates to induce mitophagy. We were able to demonstrate the accumulation of LC3-II in cells upon treatment with AMP-TPPs. The increase in LC3-II signals (Figure 7A in red), which significantly merged with mitochondrial signals (Figure 6B in green), indicated the induction of mitophagy. This effect was more pronounced in TNBC-resistant cells (Figure 7B). To confirm these results, we performed Western blot analysis and demonstrated the accumulation of the lipidized form of LC3-II presumably in resistant TNBC cells (Figure 7C).

**Figure 6:**
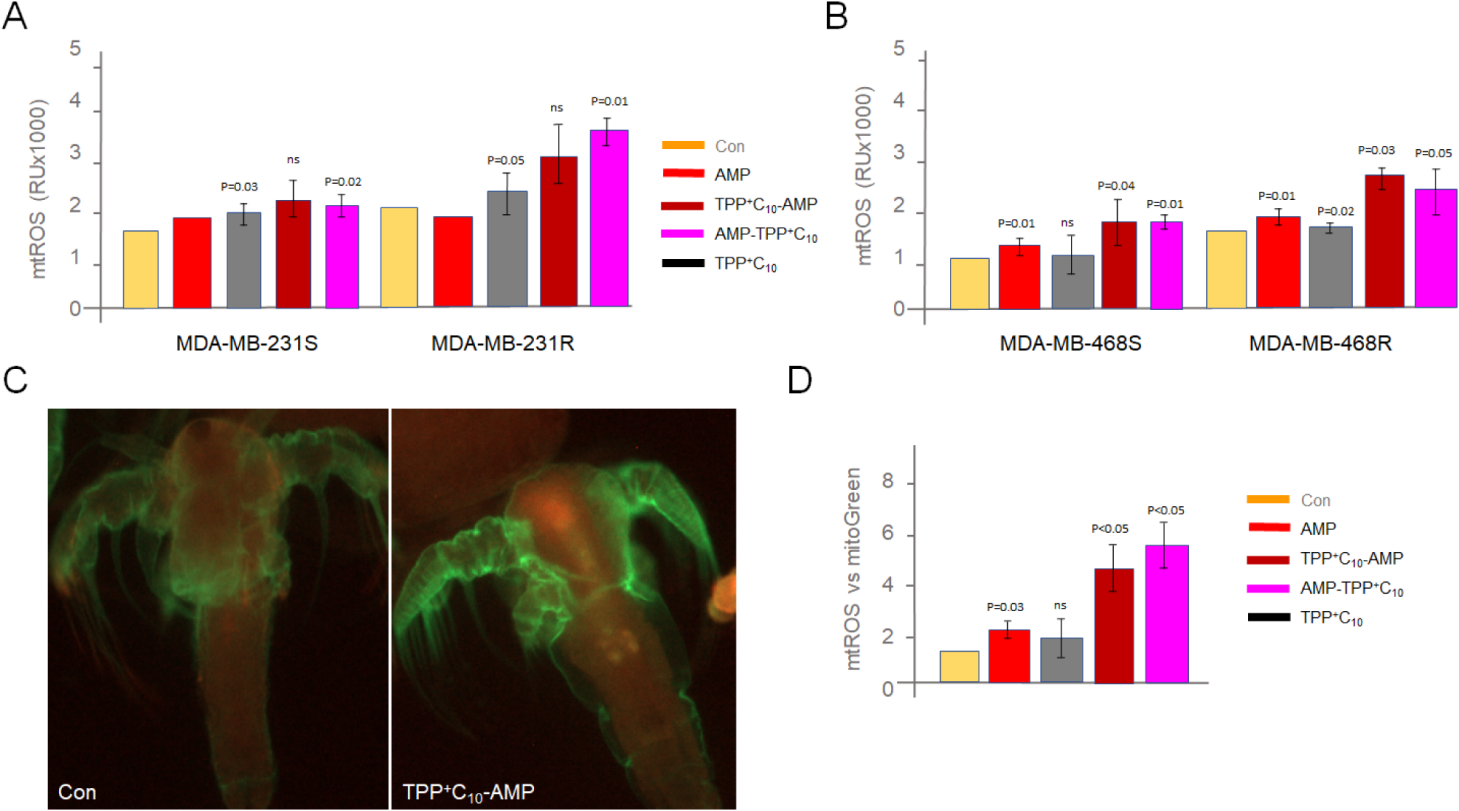
Selected AMPs induce oxidative stress in both *in vitro* and *in vivo* models. AMP conjugates and controls at IC20 were incubated with MDA-MB-231 (A) or MDA-MB-468 (B) cells. Mitochondrial ROS accumulation was measured with MitoSoxRed (*n* = 4). (C) Representative live image of *A. salina* nauplii treated or untreated with TPP-conjugated AMP for 6 h. Organisms were stained with MitoSox Red and MitoTracker Green reagents and mtROS (red) signals per species were normalized to mitochondrial content (green). (D) Results were plotted on the diagram (*n* = 3).

**Figure 7:**
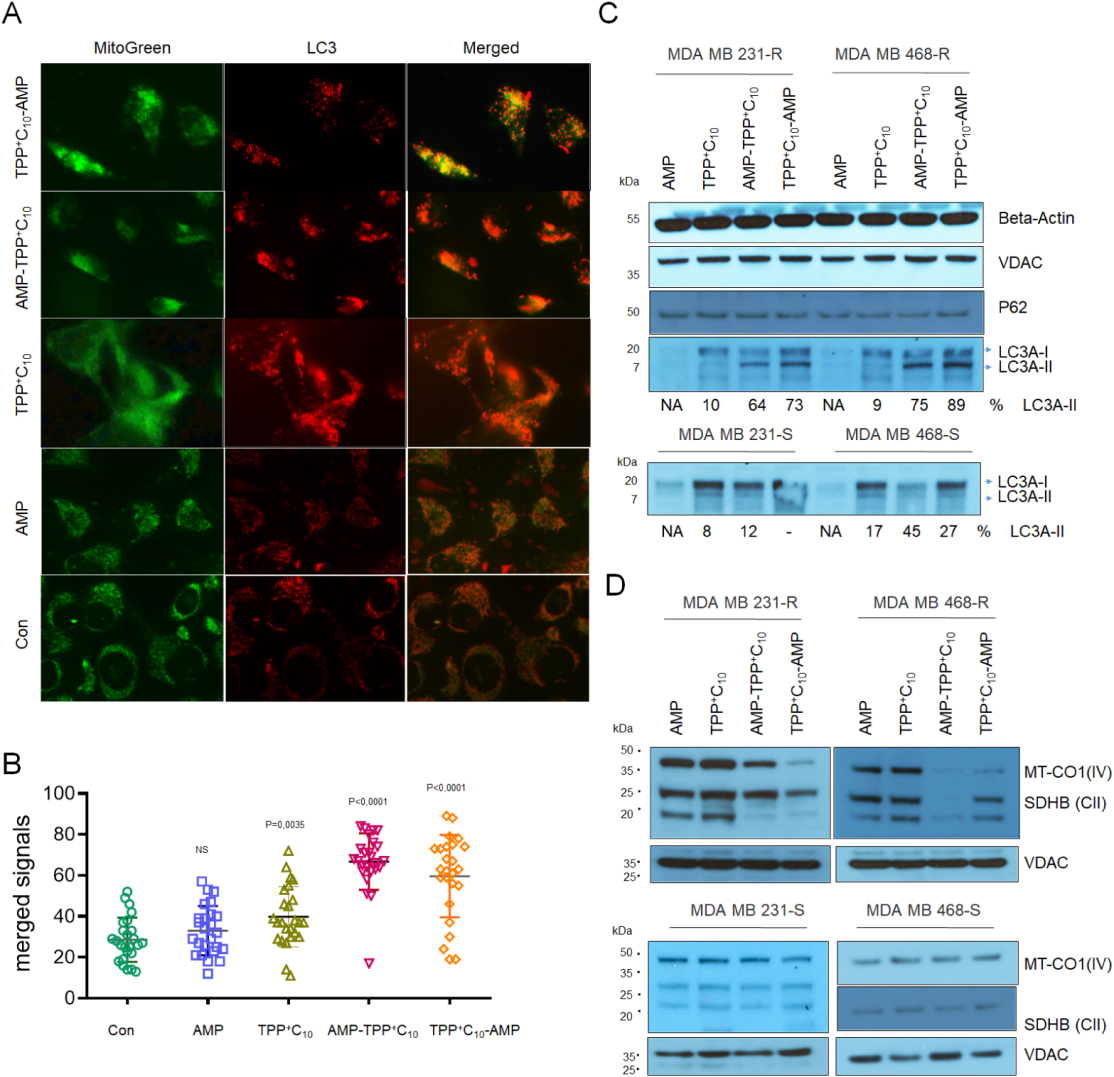
AMPs induce mitophagy and inhibit mitochondrial protein expression. (A) MDA-MB-231R cells were transfected with 1 μg of LC3-II-RFP pDNA in 6-well plates and after 2 days were treated with corresponding compounds for 1 day. After costaining with MitoTracker Green reagent, images were taken at 40× magnification and (B) results of merged red and green channels were calculated as the percentage of mitophagic events per cell (*n* = 25). (C) Results of Western blot for the autophagy marker LC3-II and p62 demonstrate increased lipidated LC3-II forms. Below are shown % of LC3-II lipidated vs nonlipidated signals normalized to VDAC signals as loading controls. (D) Shown are results of Western blot for the cytochrome c oxidase (complex IV) and SDHB (complex II) expression. VDAC is used as a loading control.

In addition, recent data on the anticancer activity of AMPs suggest mitosomal inhibition of protein synthesis.^53^ In this regard, we performed a Western blot analysis of the expression of several mitochondrial proteins after exposure to conjugated peptides. We found decreased expression of several proteins from the mitochondrial respiration complexes CIV (MT-CO1) and CII (SDHB) in the presence of AMP-TPPs predominantly in chemoresistant cells (Figure 7D), possibly indicating suppression of mitoribosomal function.

Overall, these and previous data indicated that AMPs can lead to oxidative stress in resistant TNBC cells causing mitochondrial dysfunction, which in turn can result in mitophagy and inhibition of mitochondrial protein synthesis.

## Discussion

Breast cancer is consistently ranked as the number one cause of cancer-related mortality in women with the worst prognosis in TNBC (https://www.cancer.org).^54^ Patients often relapse and develop chemoresistance, the causes of which are not fully understood,^55^ and treatment is complicated by tumor heterogeneity leading to metastasis and ultimately patient death.^2^ For this reason, there is an intense worldwide search for compounds for targeted therapies that preferentially eliminate chemoresistant forms of cancer.

Our recent studies, as well as work in other laboratories, have suggested that malignant tumors very often contain both glycolytic cells (supporting the Warburg effect), which can be destroyed by conventional chemotherapeutic agents, and OXPHOS-dependent resistant cells (contrary to the Warburg effect), which are difficult to treat and contribute to a poor clinical prognosis.^24^ The same is true for CSC populations often determining the ability of tumors to metastasize.^56^ Moreover, chemotherapy or radiotherapy preferentially selects clones resistant to OXPHOS, making the tumor even more aggressive and metastatic.^24^ Resistant TNBC used in our work as an *in vitro* model is no exception, since data from patient samples indicate that a number of mitochondrial chain proteins are, for some reason, preferentially expressed in patients with resistant forms of TNBC and apparently contribute to their survival. If so, suppression of OXPHOS may serve as one of the new tools to suppress cancer resistance, particularly when OXPHOS levels are elevated and conventional therapies are ineffective.^57,58^

Previous works, including our own, have shown that repurposing a number of antibacterial drugs to target mitochondria may be an alternative approach for eradiation of CSC and resistant cancer cells.^25,28,39,48–50,59^ In the present work, we tested TPP-conjugates of alkyl-TPP related to the sequence of Bac7 (TPP-C_10_-AMP and AMP-C_10_-TPP) that have previously shown activity against Gram-negative bacteria, affinity for the bacterial ribosome,^40^ as well as anticancer activity.^17,60^ In particular, we showed that inhibition of OXPHOS by the decapeptide RRIRPRPPYL conjugated to the TPP moiety suppresses the proliferation of OXPHOS-dependent resistant TNBC cells, with respect to sensitive counterparts. We found ROS-dependent mitophagy and suppression of mitochondrial synthesis as possible mechanisms of this inhibition. This may be due to the fact that the maximum OCR capacity in resistant TNBC cells is almost reached and AMP-induced oxidative stress is manifested to a much greater extent than in sensitive cells, resulting in increased mitochondrial dysfunction, mitophagy, and decreased mitochondrial protein synthesis.^61^ Since ROS-mediated mitophagy eventually removes dysfunctional organelles, it is conceivable that simultaneous inhibition of OXPHOS with mitophagy inhibitors may enhance the therapeutic effect of AMPs.^26^ In the current study, we also showed that it is the resistant cancer cells that have a greater potential for metastasis (increased migration and MMP activity, 3D-mammosphere formation), and it is these properties that are suppressed by the selected AMP-TPPs. This fact opens another possibility for combinatorial targeted intervention against resistant cancer cells.

In addition to the above-mentioned mechanisms, we found suppression of the synthesis of some mitochondrial respiratory chain proteins, and therefore, it remains an open question what other mechanisms contribute to the suppression of chemoresistant cell proliferation through AMP-TPPs, in particular, what may be the role of mitochondria in such inhibition. A number of previous studies have shown that antibiotics in the form of short peptides can involve mitochondria indirectly, for example, through the expression of some mitochondria-related apoptosis proteins.^62,63^ For example, the anticancer activity of MSP-4 in osteosarcoma cells is associated with the induction of apoptosis through activation of Fas/FasL- and intrinsic mitochondria-mediated pathways.^64^ Other AMPs like Dermaseptin-PS1,^18^ Epinecidin-1,^65^ or CM4 ^22^ exhibit anticancer activity through disrupted cell membranes, which again is tied to apoptosis. Only a few recent works, including ours, have shown that oxidative stress induced by AMPs can lead to mitochondrial dysfunction and somehow reduce cancer cell proliferation.^66,67^ For now, our working hypothesis is that AMPs can penetrate organelles and affect OXPHOS either directly via mitoribosomal translation or indirectly, by inducing oxidative stress followed by mitochondrial dysfunction and mitophagy.

Stability and mitochondrial selectivity of AMP conjugates remain a challenging issue. Beginning with Dr. Murphy’s foundational work,^68^ TPP-linked compounds have demonstrated efficient and rapid mitochondrial delivery via membrane potential-driven accumulation, with retention sufficient for biological action even under partial proteolysis. Murphy’s group validated the mitochondrial targeting and stability of TPP^+^C10 conjugates,^69,70^ the same length linker used in our study. Additional studies confirm that TPP enhances peptide stability and mitochondrial accumulation,^71,72^ and that conjugates reach mitochondria within seconds—the only negatively charged organelles. While short AMPs degrade rapidly in serum^73^ (e.g., Bac7, *t*_1/2_ < 2 h), modifications including TPP conjugation significantly prolong their half-life^74^ and support mitochondrial targeting.^75^ Our current data show that TPP-C10 alone has minimal impact on proliferation, with mild mitochondrial depolarization (Figure S3C,D), whereas AMP activity is enhanced by TPP conjugation. Importantly, mitochondrial dysfunction is selectively induced in resistant, OXPHOS-dependent cancer cells without broadly affecting normal cells. Finally, post-targeting cleavage of TPP-peptide conjugates may further increase localized activity within mitochondria.^76^ Given the published evidence on stability and localization, the observed cellular and *in vivo* effects seem to reflect the action of the intact TPP-AMP conjugate rather than the free fragments. Either way, with a reasonable approach and improved stability and delivery methods of small peptides, we believe that it will be possible to target certain types of resistant tumors with elevated levels of OXPHOS, which promises certain clinical benefits.

## Conclusions

Short antimicrobial peptides, particularly when conjugated with the mitochondrial-targeting TPP moiety, effectively inhibit the metastatic potential of chemoresistant triple-negative breast cancer cells. These compounds not only reduce the formation of CSC-like mammospheres but also exert anticancer effects through the induction of mitochondrial dysfunction, characterized by impaired mitochondrial protein translation, elevated oxidative stress, and enhanced mitophagy. These findings support their potential as targeted therapeutics against resistant and aggressive cancer phenotypes.

## Supplementary Material



## Notes

Additional experimental procedures and figures are provided in the Supporting Information (PDF)

## Abbreviations

TPP: triphenylphosphonium
FCCP: carbonyl cyanide-*p*-trifluoromethoxyphenylhydrazone
ΔΨM: mitochondrial membrane potential
OCR: oxygen consumption rate
COX: cytochrome c oxidase
CSC: cancer stem cells
mtDNA: mitochondrial DNA
MMPs: matrix metalloproteases
TNBC: triple-negative breast cancer
OXPHOS: oxidative phosphorylation
ROS: reactive oxygen species
