# Supplementary material for: Toward Mitochondrial Targeting of Resistant Triple-Negative Breast Cancer Using Triphenylphosphonium-Conjugated Antimicrobial Peptides
Source: ACS Pharmacol Transl Sci. 2025 Oct 7;8(11):4159–71. doi: 10.1021/acsptsci.5c00563 (PMC12624430; doi:10.1021/acsptsci.5c00563)
Supplement: Supplementary file 1 [file pt5c00563_si_001.pdf]

Supporting Information

TOWARDS MITOCHONDRIAL TARGETING OF RESISTANT TRIPLE-NEGATIVE BREAST CANCER USING TRIPHENYLPHOSPHONIUM-CONJUGATED ANTIMICROBIAL PEPTIDES

Eda Kapan[1]‡, Cemile Uslu[1]‡, Haya Arab[1,2], Leen Ahmed[1], Rama Ali[1], Andrey G. Tereshchenkov[3], Natalia V. Sumbatyan[4], and Alex Lyakhovich[1]*

[1]Sabanci University, Molecular Biology, Genetics and Bioengineering, Faculty of Engineering and Natural Sciences, Üniversite Caddesi No: 27, 34956 Orta Mahalle, Tuzla – İstanbul, Turkey;

[2]Trinity College Dublin, the University of Dublin, College Green, Dublin 2, D02 PN40 Ireland;

[3]Lomonosov Moscow State University, A.N. Belozersky Institute of Physico-Chemical Biology, 1/40 Leninskie Gory, 119234 Moscow, Russia;

[4]Lomonosov Moscow State University, Department of Chemistry, 1/3 Leninskie Gory, 119991 Moscow, Russia;

‡ equal contribution;

*Sabanci University, Faculty of Engineering and Natural Sciences, Office FENS1043, Üniversite Caddesi No: 27, 34956 Orta Mahalle, Tuzla – İstanbul, Turkey; Email: alex.lyakhovich@sabanciuniv.edu or lyakhovich@gmail.com

Table of contents

page	Figure number	Title
S-3	Figure S1	OXPHOS-dependence of chemoresistant TNBC cells
S-4	Figure S2	Cell survivability assay for TNBC upon treatment with AMP conjugates
S-5	Figure S3	The effect of high doses of AMP conjugates on normal cells and cancer cells
S-6	Figure S4	Effect of AMP conjugates on mitochondrial content and membrane polarization
S-7	Figure S5	Effect of AMP conjugates on cell adhesion
S-8	Figure S6	Original images of wound healing assay to distinguish cell migration from cell proliferation

OXPHOS-dependence of chemoresistant TNBC cells

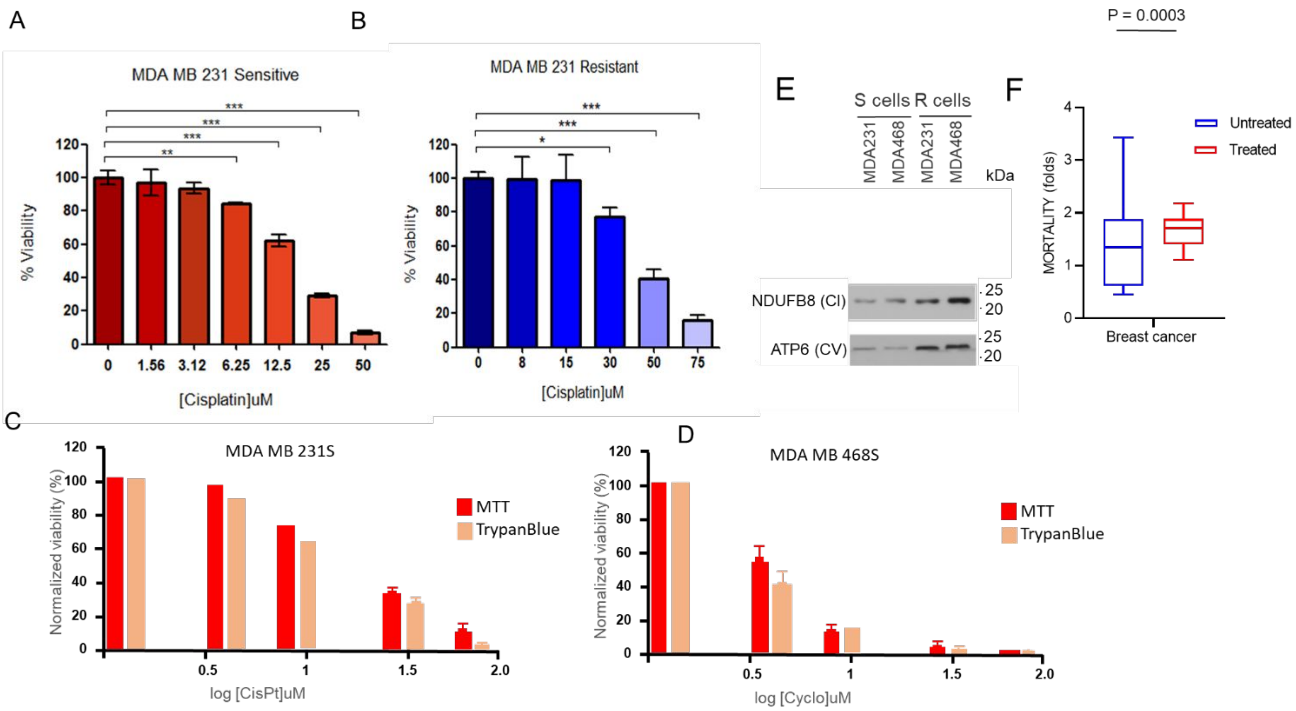

Figure S1. OXPHOS-dependence of chemoresistant TNBC cells. Cell survivability assay for TNBC and sensitive (A,C) and chemoresistant (B,D) MDA MB 231 and MDA MB 468 cells were performed by two methods – MTT assay (mitochondrial activity) and TrypanBlue staining (the dye stains dead cells). All results are representative of at least three independent experiments with at least three replicas per treatment point. Data indicate the mean ± SEM. (E) Corresponding protein profiling of mitochondrial OXPHOS proteins by Western blotting from chemoresistant (R) and chemosensitive (S) TNBC cells showing increased OXPHOS-dependence in chemoresistant cells. Loading was normalized by mitochondrial VDAC protein. (F) Inverse dependence of TNBC cancer patient survivability un/treated with chemotherapy and the expression of OXPHOS genes (n = 5143).

Cell survivability assay for TNBC upon treatment with AMP conjugates

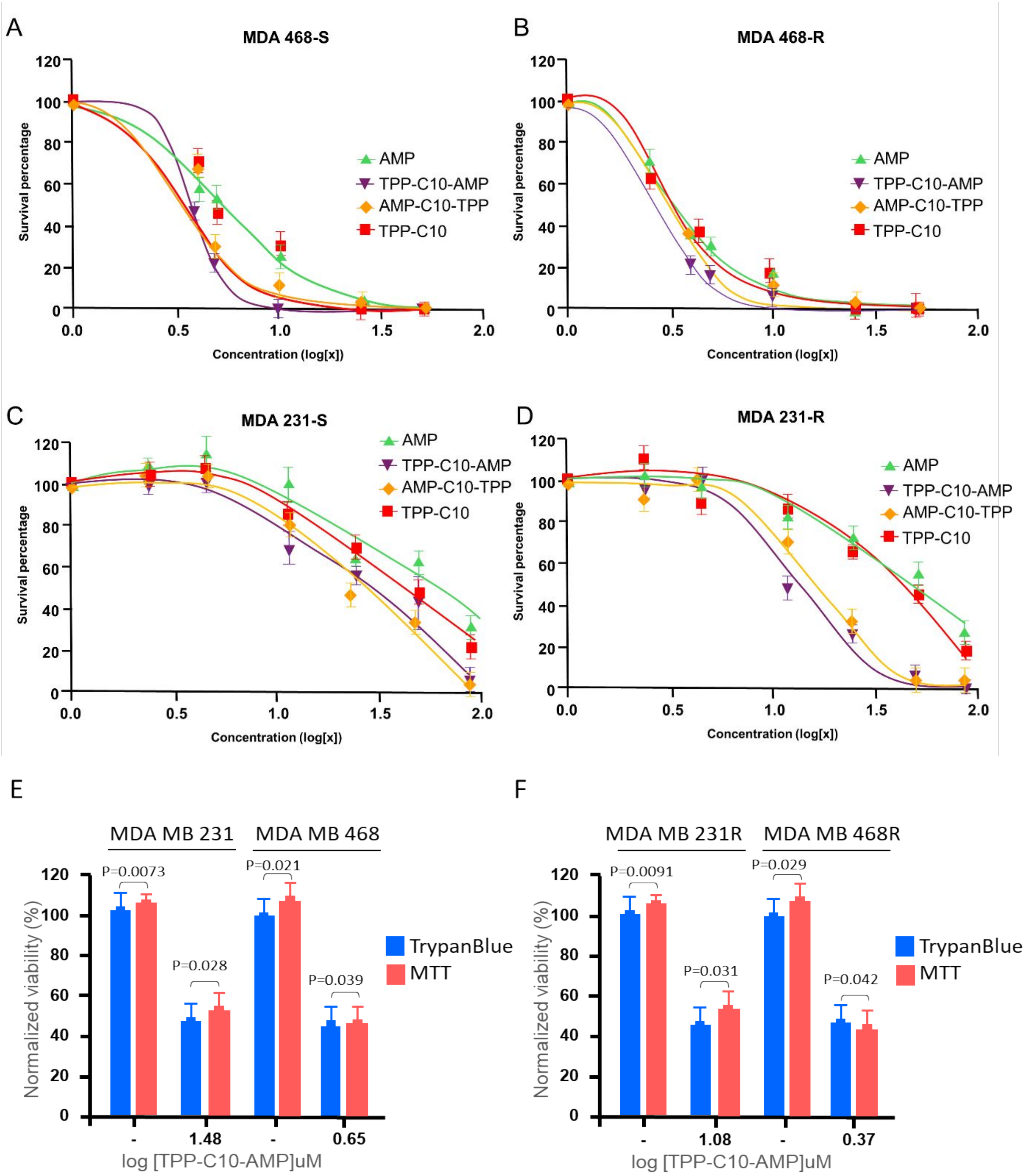

Figure S2. Cell survivability assay for TNBC upon treatment with AMP conjugates. TNBC sensitive (A,C) and chemoresistant TNBC cells (B,D) were treated with escalating concentrations of corresponding compounds for three days followed by MTT assay. All results are representative of at least three independent experiments with at least three replicas per treatment point. The exact IC50 values are provided for each graph and shown in Figure 1. Data indicate the mean ± SEM. (E,F) Cell survival analysis following treatment with TPP-C10-AMP for chemoresistant and sensitive TNBC cells was also measured using manual counting (TrypanBlue staining) and MTT assay results (n=4) to show that the results are comparable.

The effect of high doses of AMP conjugates on normal cells and cancer cells

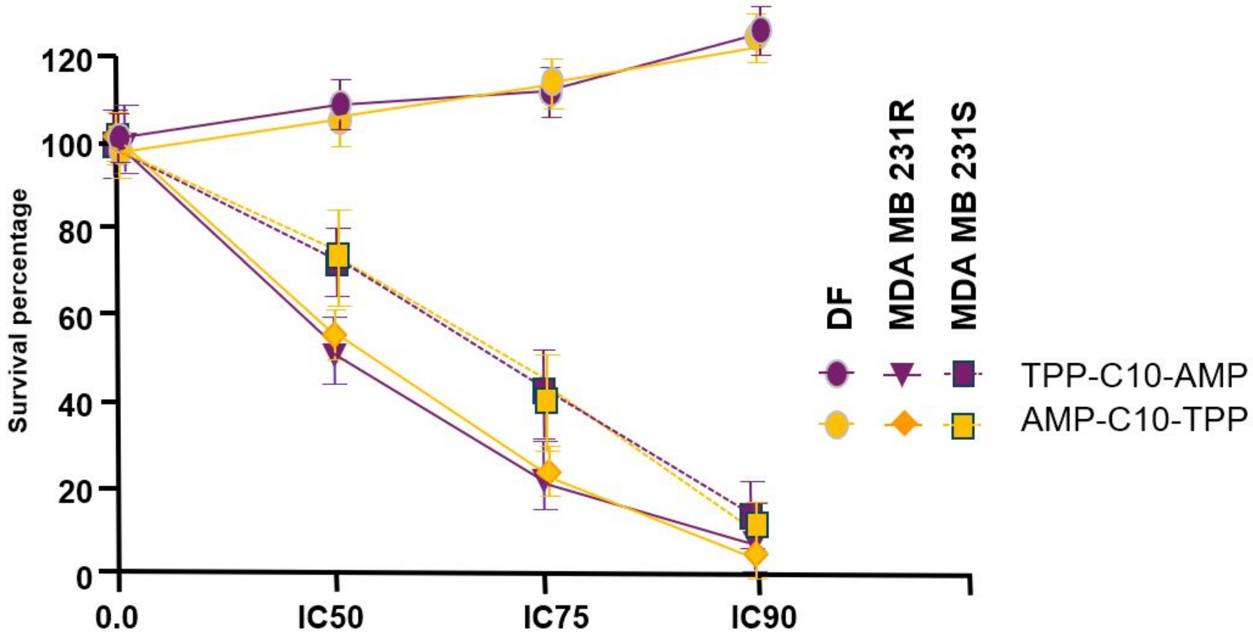

Figure S3. Comparison of cell survival at higher doses of AMP conjugates. MB 231 sensitive and chemoresistant cells as well as non-malignant dermal fibroblasts (DF) were treated with IC50, IC75 and IC90 concentrations of corresponding compounds for three days followed by manual counting by TrypanBlue excision assay. Data indicate the mean ± SEM (n=4), all $P<0.05$

Effect of AMP conjugates on mitochondrial content and membrane polarization

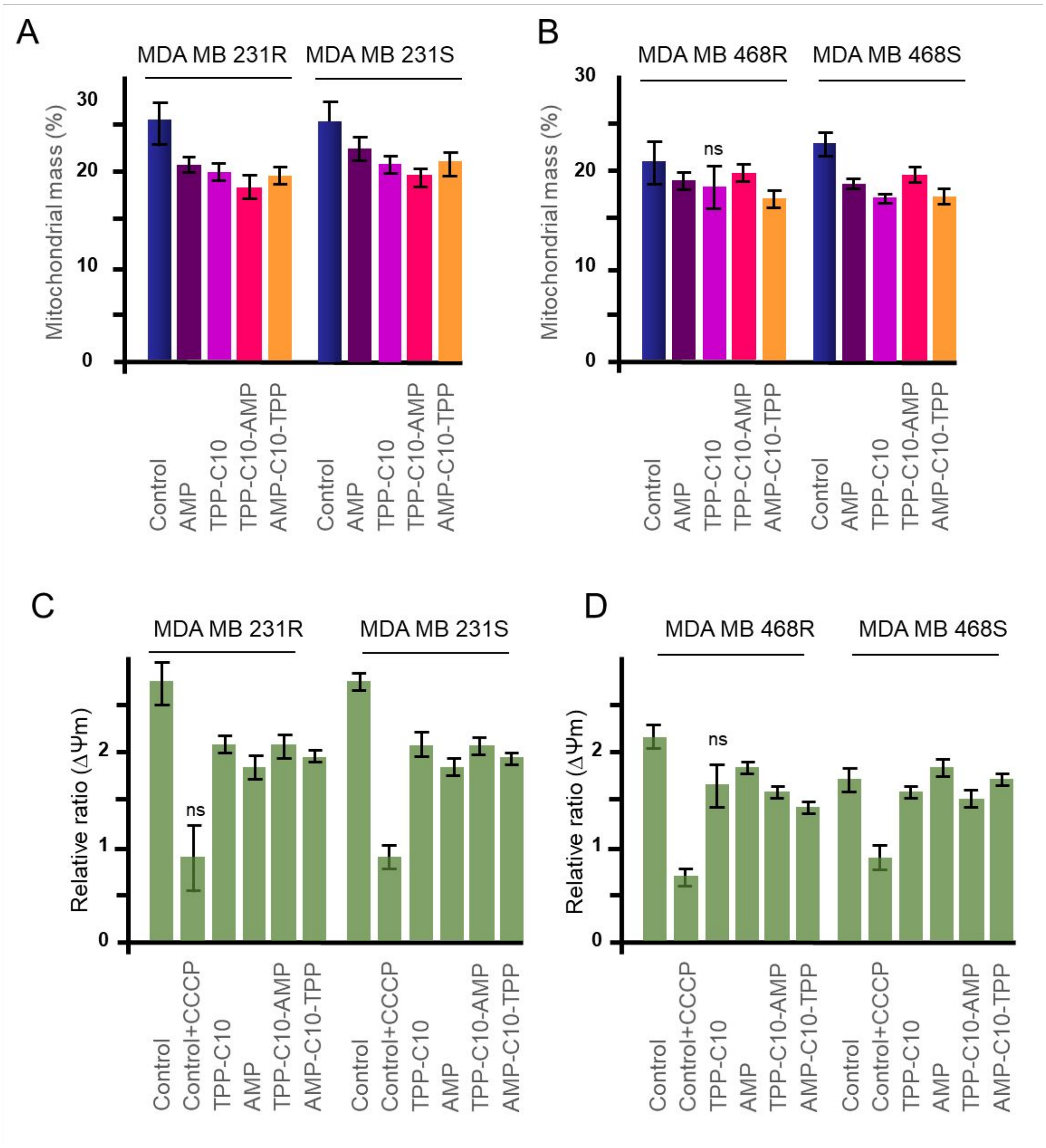

Figure S4. Effect of AMP conjugates on mitochondrial content and membrane polarization. (A,B) 10^5 cells per well in 6-well plates were pretreated with the compounds for 3 days. After staining for 10 min with MitoTracker Green cells were washed and the medium was replaced with PBS followed by fluorescence measurement at excitation/emission wavelength 490/523 nm. Results are plotted on the graph and Student's t-test applied (n=4). All calculated P values were less than 0.05. (C,D) JC-1 staining was performed to measure mitochondrial membrane potential (ΔΨm). The average red and green intensity values in each biological replica were determined and the red and green intensity ratio for each was calculated followed by Student's t-test (n=4). As a control, carbonyl cyanide 3-chlorophenylhydrazone (CCCP) was added 0.2 µM 6 h prior to JC1 staining. NS-non significant.

Effect of AMP conjugates on cell adhesion

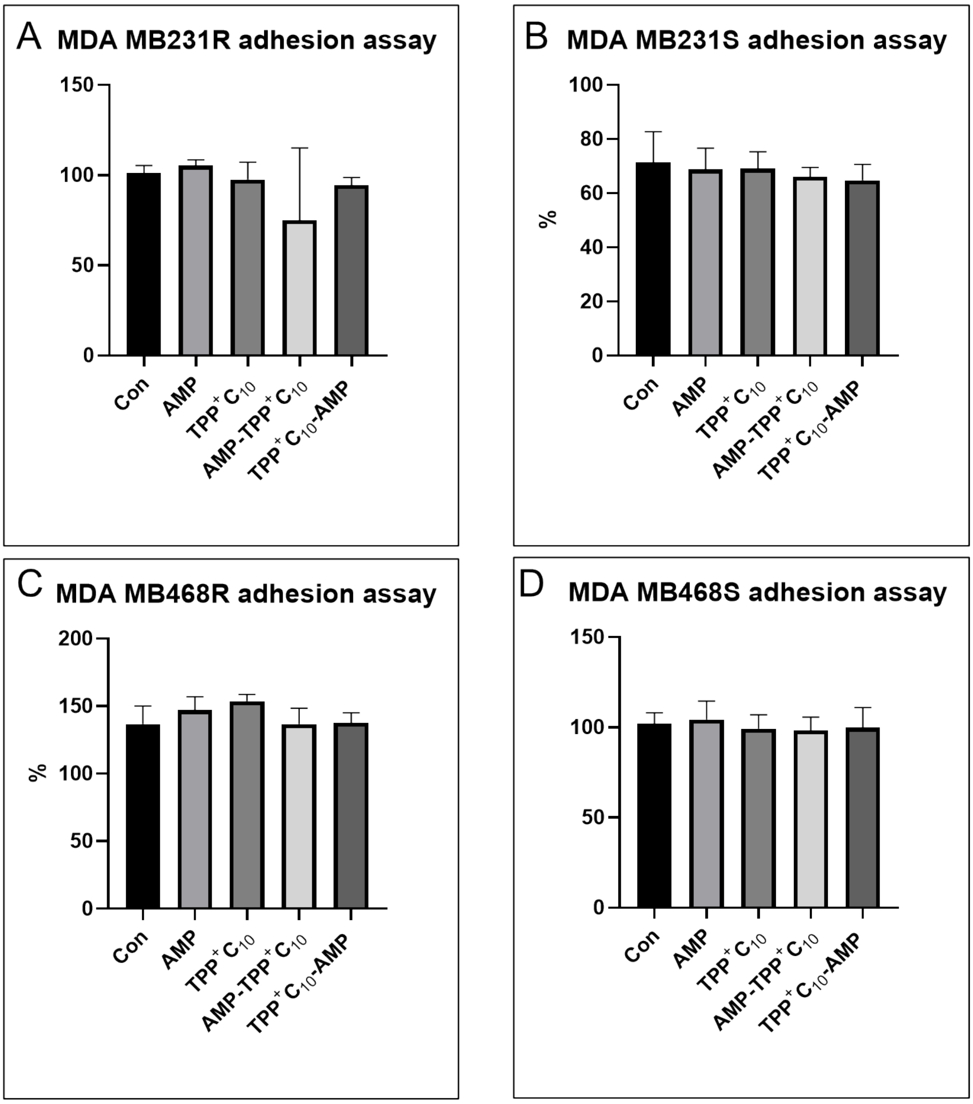

Figure S5. Effect of AMP conjugates on cell adhesion. 10^5 cells per well in 6-well plates were pretreated with the compounds for 1 day and adhesion was assessed as described in Materials and Methods. Adhered cells were cross-linked with paraformaldehyde, stained with crystal violet, and after addition of lysis buffer with ethanol, total OD450 was counted and plotted on bar graphs. Adhesion assay for resistant (A,C) and sensitive (B,D) The data are shown as % of adherent cells normalized to untreated (control) MDA MB 231 cells. All values were significant ($P<0.05$). N=4.

Original images of wound healing assay to distinguish cell migration from cell proliferation

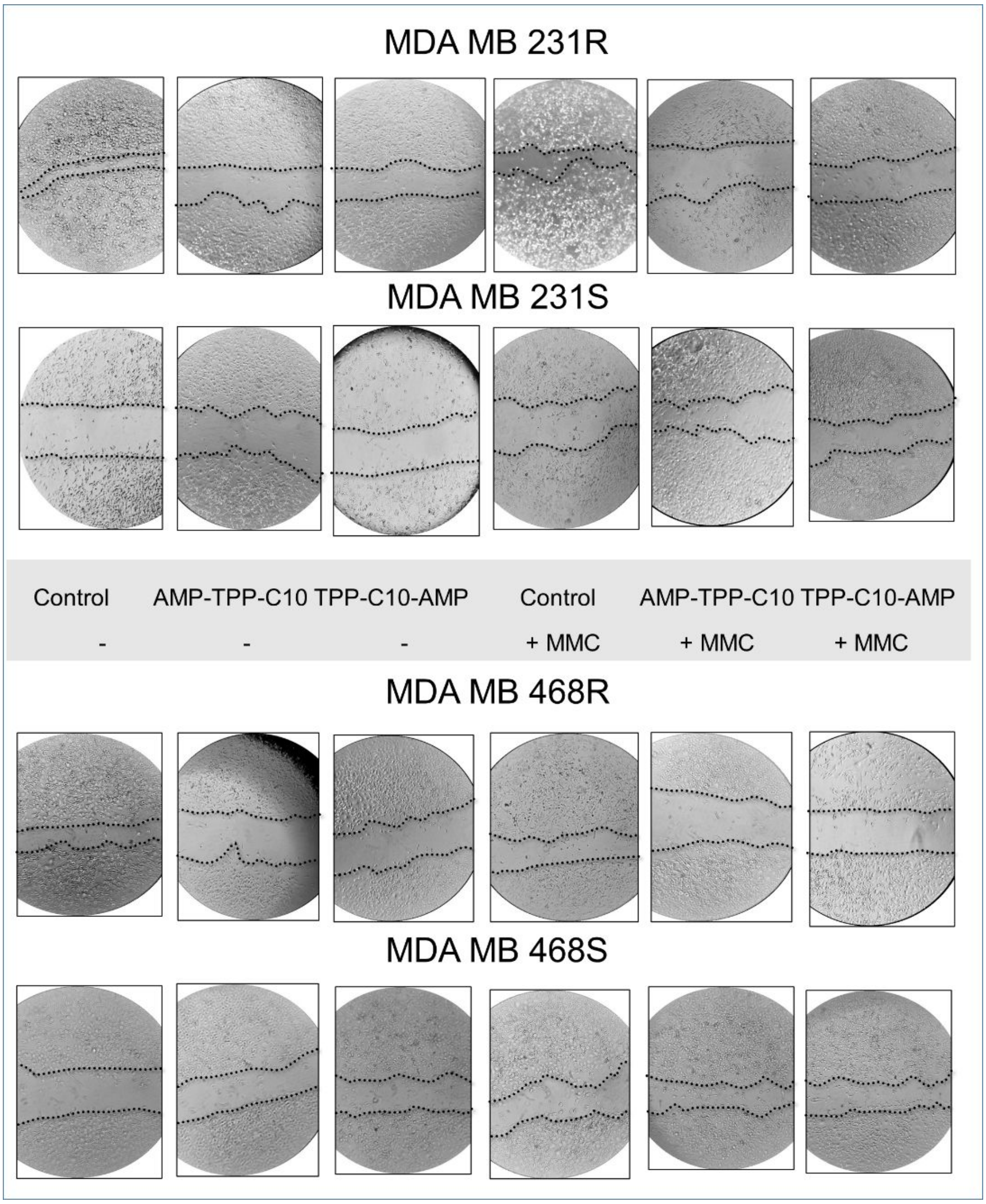

Figure S6. Original images of wound healing assay to distinguish the effects of TPP conjugates on cell migration from cell proliferation. MDA MB 231 and MDA MB 468 TNBC cells in 6-well plates at a cell density of 80% were treated with IC20 of the TPP conjugates in the presence of the antiproliferative compound mitomycin C (0.0002 mg/ml, MMC), which blocks DNA replication. Migration was assessed 36 h after scratch application and the gap-filling rate was calculated and plotted on the graph in Figure 3 E,F.
